# Evaluation of economic burden with biologic treatments in Crohn’s disease patients: A mirror image study using an insurance database in Japan

**DOI:** 10.1371/journal.pone.0254807

**Published:** 2021-07-19

**Authors:** Celine Miyazaki, Nagano Katsumasa, Kuan Chih Huang, Yan Fang Liu

**Affiliations:** 1 Health Economics Department, Janssen Pharmaceutical K.K., Tokyo, Japan; 2 Medical Affairs Department, Janssen Pharmaceutical K.K., Tokyo, Japan; 3 Janssen Research & Development Epidemiology, Taipei, Taiwan; 4 Global Epidemiology, Janssen Research & Development, Singapore, Singapore; University of South Carolina, UNITED STATES

## Abstract

Biologics are recommended in Japan to treat moderate to severe Crohn’s Disease (CD). Although CD is associated with high direct costs in Japan, updated information after ustekinumab’s approval is unavailable. We aimed to evaluate the healthcare resource utilization (HRU) and associated direct costs from the payer’s perspective in Japan. Claims data (2010–2018) were retrospectively analyzed to identify patients with CD. HRU and associated costs were evaluated for 12 months before and after biologic initiation and followed-up till 36 months post-initiation. Outcomes were reported using descriptive statistics. Among the included patients (n = 3,496), 1,783 were on biologics and 1,713 were on non-biologics. Mean (SD) age was 36.4 (13.2) years and patients were predominantly male (76.1%). Patients aged 18–39 years were affected with CD the most (55.3%). Biologic initiation was associated with a reduction in inpatient stay, length of stay, outpatient visits, and associated costs; and an increase in pharmacy costs and total costs after 12 months. Extended follow-up showed a decreasing trend in HRU and costs till 24 months but an increase after 36 months. These findings demonstrated reduction in clinical burden and slight increase in economic burden with biologics. However, indirect costs also need to be evaluated.

## Introduction

Crohn’s disease (CD) is a chronic, progressive, inflammatory gastrointestinal disease leading to bowel damage and disability due to complications such as bowel strictures, abscesses, or fistulas [1, 2]. The number of individuals affected with CD in Japan was reported to be 40,000 in 2013 by the Japan Intractable Diseases Information Center [3] and 70,700 in 2014 based on a nationwide survey by Murakami et al [4]. Additionally, the prevalence of CD in Japan has been increasing with time from 0.88 per 100,000 in 1965 [5] to 55.6 per 100,000 in 2014 [4].

As the goal of CD treatment is prevention of progression, symptom control, and reduction in risk of complications, effective long-term management and treatment persistence is essential [6]. The standard of care for CD in Japan includes corticosteroids, enteral nutrition therapy, 5-aminosalicylic acid (5-ASA), immunomodulators, biologics, and surgery depending on disease severity and presence/absence of complications [7, 8]. Biologics are recommended for patients with moderate to severe CD, if steroids and/or nutritional therapy is not effective [7]. Infliximab was the first biologic to be approved for CD by Japan in 2002 [9], followed by adalimumab in 2010 [10] and ustekinumab in 2017 [11]. The advent of these biologics have led to improved long-term prognosis; reduction in relapse rate, requirement of surgery, and issues related to steroid discontinuation; as well as enabled patients to receive treatment in an outpatient setting [7, 12, 13].

The introduction of the Intractable Disease Health Care Act in Japan allowed patients with rare diseases such as CD to receive financial assistance in lieu of authorizing usage of their data for research [14]. Such subsidies are provided to patients with a predetermined severity level as defined by the disease severity classification and who face impediments in their daily life [15]. However, as 331 diseases including CD are covered under the Act as of 2018 [15], balancing adequate healthcare coverage with available funds is a challenge. Moreover, the rapidly aging population (27.7% aged >65 years) and simultaneous reduction in labor force has strained the healthcare sector, since taxes obtained from working individuals for public health programs are inadequate to sustain coverage for the whole population [16].

As advanced medications become available to meet the unmet medical needs of patients with CD, there is concern whether excessive prescriptions in real-world treatment practice in Japan could have a major impact on the direct costs associated with CD. Previous Japanese studies have reported high healthcare resource utilization (HRU) and costs associated with CD [17, 18]. While Yamabe et al [17] analyzed data from the 2012–2014 Japan National Health and Wellness Survey and reported mean annual per-patient direct costs to be ¥7,533,257; another claims data analyzed by Saito et al [18] from 2013–2016 reported median per-member per-year direct costs to be ¥1,957,320. Since ustekinumab was approved for CD in Japan in 2017 [11], the aforementioned studies lack information on its impact on clinical and economic burden. It is not only essential to understand the impact of advanced treatments on clinical conditions of CD, but also the impact on economic burden. Hence, in the current study we aimed to evaluate HRU and direct costs among Japanese patients on biologic therapy (including ustekinumab) for the treatment of CD.

## Methods

### Data source and study design

**Fig 1:**
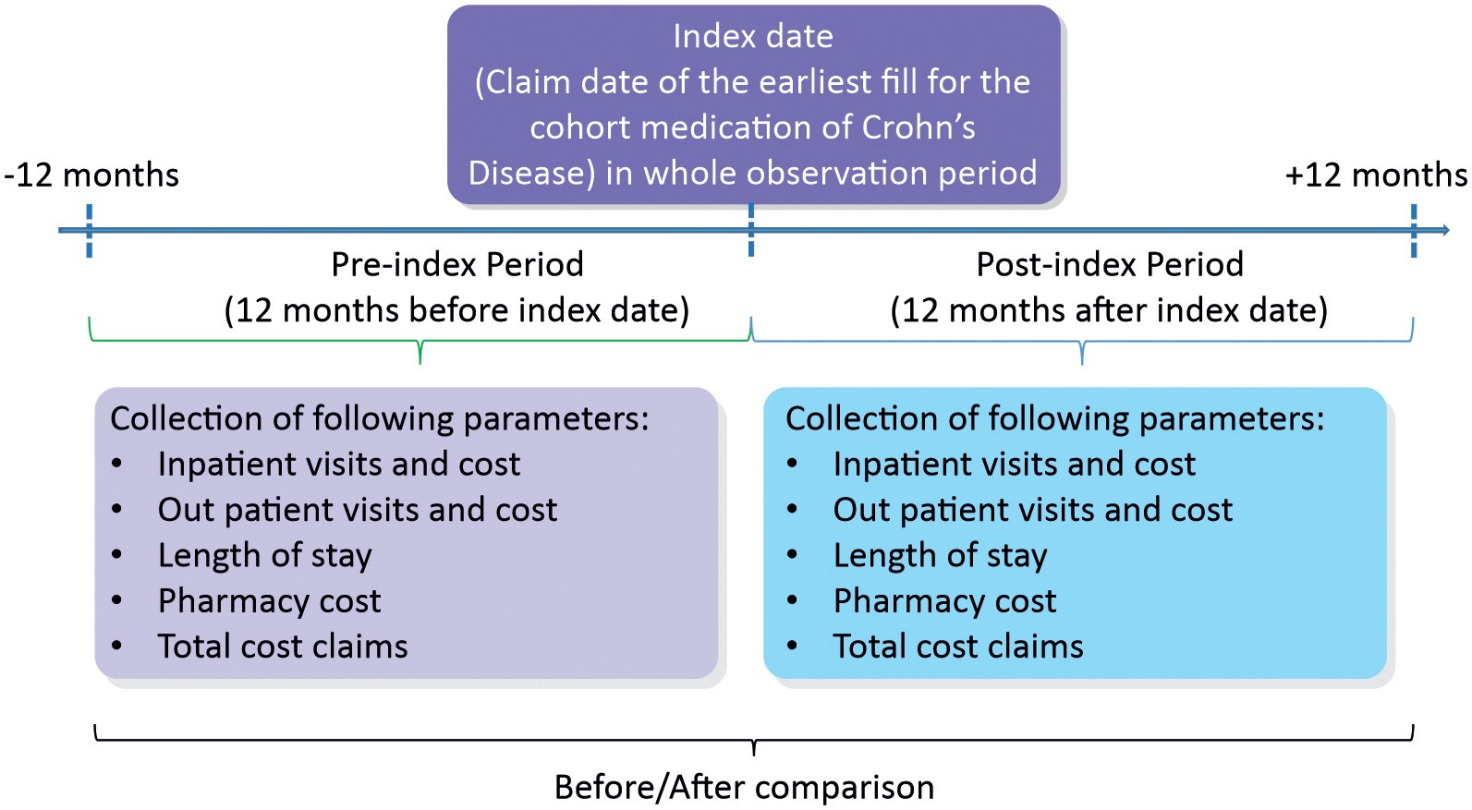
Mirror image study design.

A mirror-image design was used for this study focusing on pre-post comparisons of medical resource used and claims cost estimate of one year before and after the biologic index date to understand the economic burden of biologics from the payer’s perspective in Japan (Fig 1) Retrospective claims data obtained from the Japan Medical Data Center (JMDC) database from 01 January 2010 to 30 September 2018 were analyzed. The database comprises corporate employees from medium to large scale companies and their dependents, and has been capturing inpatient, outpatient, pharmacy, and medical examination data for approximately 4.2 million individuals (at the time of analysis) since January 2005 [19]. The database has been widely used in health services research [20–22]. As long as an individual belongs to the Health Insurance Association, their medical consultation information can be tracked longitudinally, even if they attend multiple medical institutions or transfer to another hospital.

Information retrieved from the JMDC database was anonymized to protect patient privacy under the “Act on the Use of Numbers to Identify a Specific Individual in the Administrative Procedure”. This study was approved by the sponsor’s internal approval committee in accordance with Japanese ethical and legal guidelines, Inflammatory Bowel Disease Research Concept Approval Team and Health Economics Review Board. The informed consent of patients was not required as per the Ethical Guidelines for Epidemiological Research issued by the Japanese Ministry of Health, Labor, and Welfare (MHLW), since the analysis were conducted on the retrospective anonymized claims database managed by JMDC. Reporting of claims data was based on International Society for Pharmacoeconomics and Outcomes Research guidelines [23].

### Patient selection

**Fig 2:**
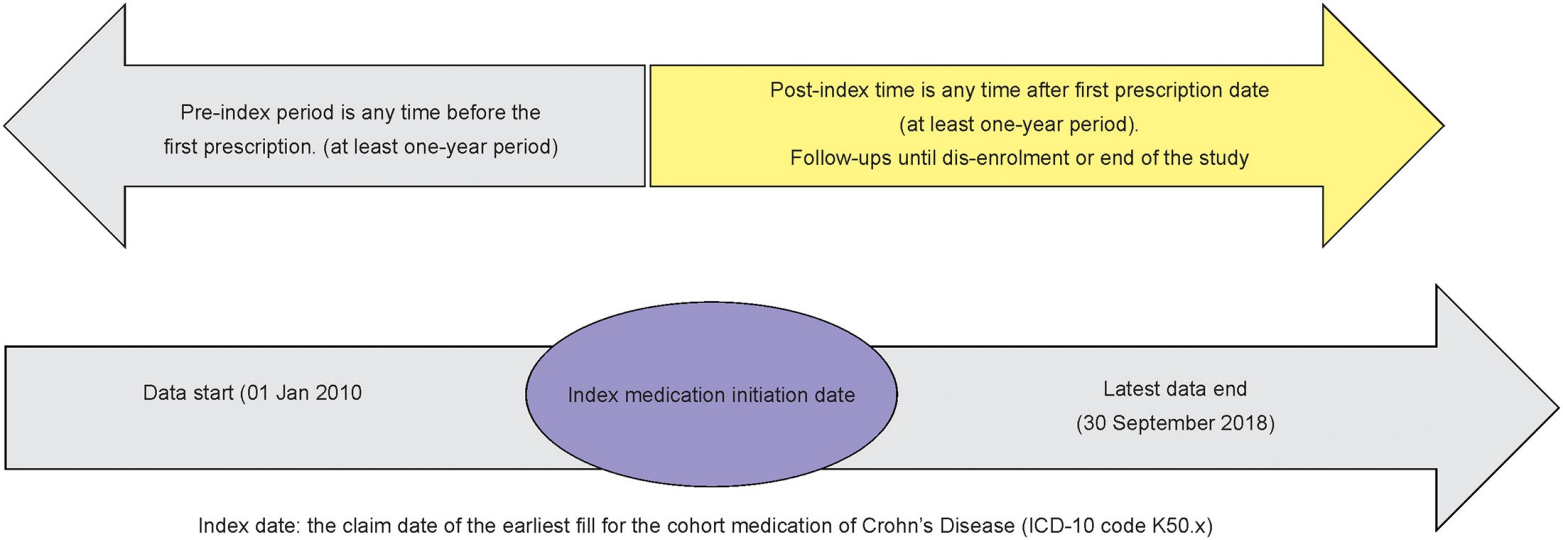
Study framework. Index date: the claim date of the earliest fill for the cohort medication of Crohn’s Disease (ICD-10 code K50.x).

Patients with a diagnosis of CD (International Classification of Diseases, 10^th^ revision Clinical Modification K50.x) records within the timeframe of the evaluation period; at least one record of CD treatment (e.g. pharmacological agents, gastrointestinal procedure, enteral nutrition procedure and surgical procedure [S1–S3 Tables]) from the first CD diagnosis date; and 12 months of insurance coverage before and after the index date were included into the study (Fig 2). Since the highest incidence of CD is in the mean employment age, the current study uses claims data which is most appropriate to track the cost of illness and evaluate the cost driver to patients. Claims data can track the overall direct healthcare cost even when the patient is shifted to another hospitals or uses multiple facilities. Patients were followed-up within the timeframe period from the index date (earliest fill for CD-related medication) until dis-enrolment or end of study period whichever occurred first. Patients who did not have a record of CD-related treatment [24] after the CD diagnosis date were excluded.

Eligible patients were categorized into a non-biologic group i.e. patients without a biologic treatment record (e.g. 5-ASA, corticosteroids, and immunosuppressants) and a biologic group i.e. patients with a biologic treatment record (e.g. adalimumab, infliximab, and ustekinumab) (S1 Table); and further stratified by age as adults (≥18 years old) or children (<18 years old). The patients were also segregated as per their insurance status into employees (adults), adult dependents, and children. This was performed because severe CD would hinder an employee’s ability to work, hence affecting enrolment status in the database. Employees who were enrolled were representative of the workforce, while adult dependents and children were representative of the general population.

### Study measures

Claims information such as demographics (age and gender), insurance status, clinical characteristics (Charlson Comorbidity Index [CCI] and comorbid conditions), diagnosis (anatomical location of CD), dispensed drugs, gastrointestinal surgical interventions, and hospitalization records of patients were extracted from the database for analysis.

### Healthcare resource utilization and cost evaluation

HRU measures such as outpatient visits, inpatient visits, surgery, enteral nutrition, and pharmacy claims, and cost claims associated with CD were obtained from the JMDC database. The medical resource used and cost estimate of one year before the biologic index date (first claim record of a biologic) and one year after the biologic index date was evaluated by the respective HRU categories. All costs were reported as Japanese Yen (¥) and were not adjusted for inflation because rates and prices have been flat for the past decade in Japan [25].

The cost of each resource was also reported over a follow-up period of 12, 24, and 36 months from the defined biologic index date. Biologic-prescribed patients with a continuous claims record for 12, 24, or 36 months were included in this analysis. For each resource category, the sum of costs of patients were divided by the sum of their follow-up time in months. Data were obtained only if the patients were in the health plan throughout the observation period.

### Statistical analysis

Data for all study measures were summarized descriptively as mean values, SD, median, and range for continuous variables and frequency distribution for categorical variables. Baseline demographics and clinical characteristics were presented according to their assigned treatment group. Comorbidities were assessed with the help of CCI prior to the index date for biologic users and throughout the entire enrolment period in JMDC for non-biologic users. CCI categorizes comorbidities of patients and has an associated weight (1 to 6), on the basis of adjusted risk of mortality or resource use, sum of all the weights provides a single comorbidity score for a patient. A score of zero means no comorbidities and higher the score, the more likely the predicted outcome will result in mortality or higher resource use. Overall cost of CD includes following parameters: inpatients (any medical resource charges that is related to hospital admission and direct primary care hospital cost), outpatients (medical resource charges at outpatient visits and visiting fees) and pharmacy cost. CCI and average costs per patient per month, by the type of medical resource utilization used (inpatients, outpatients, pharmacy), were reported as mean (SD) or median (interquartile range [IQR], which is the difference between the third and first quartiles).

## Results

### Patient demographics

**Fig 3:**
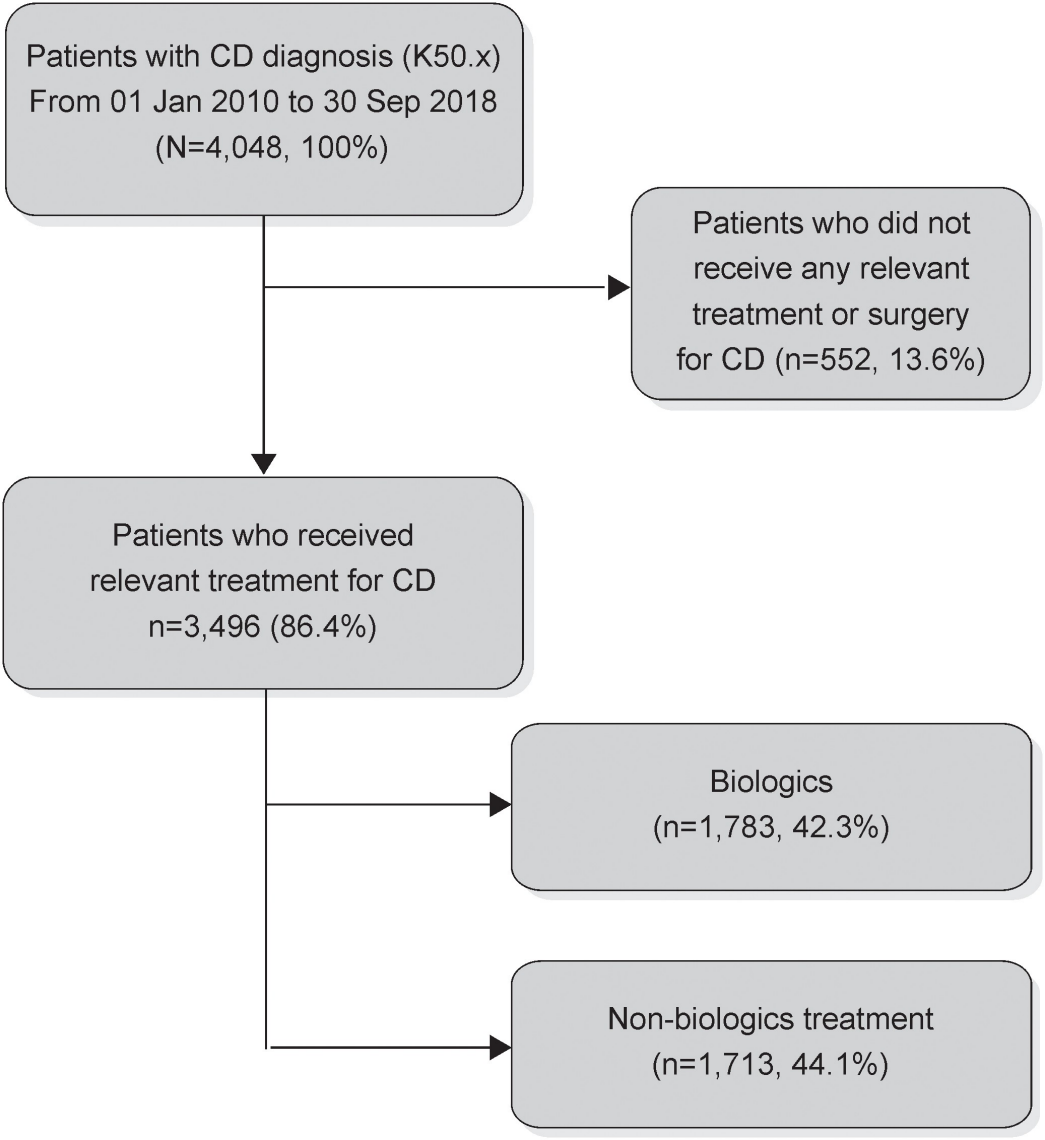
Selection flow chart.

After applying the inclusion and exclusion criteria, the included patients with CD (n = 3,496) were separated into two groups, biologic-treated (n = 1,783) and non-biologic treated (n = 1,713) (Fig 3).

The mean (SD) age of the overall cohort was 36.4 (13.2) years and majority of the patients were male (76.1%). Majority of the claims were from employed patients (72.3%), followed by dependent adults (21.6%), and children (6.1%). Many patients were also affected by comorbid conditions such as peptic ulcer disease (47.9%), chronic pulmonary disease (38.3%), and mild liver disease (24.6%) (Table 1).

**Table 1:** Demographics and characteristic of patients with CD.

	Non-biologics* (n = 1,713)	Biologics (n = 1,783)	All patients (n = 3,496)
**Age, years (mean [SD])**	38.8 (14.1)	34.2 (11.8)	36.4 (13.2)
**Age category, years (n [%])**			
< 18	99 (5.8)	115 (6.4)	214 (6.1)
18–29	414 (24.2)	615 (34.5)	1029 (29.4)
30–39	402 (23.5)	502 (28.2)	904 (25.9)
40–49	383 (22.4)	366 (20.5)	749 (21.4)
50–59	295 (17.2)	151 (8.5)	446 (12.8)
60–69	109 (6.4)	34 (1.9)	143 (4.1)
≥ 70	11 (0.6)	0 (0)	11 (0.3)
**Gender, n (%)**			
Male	1,254 (73.2)	1,405 (78.8)	2,659 (76.1)
Female	459 (26.8)	378 (21.2)	837 (23.9)
**Location of CD, n (%)**			
K50.0—small intestine	236 (13.8)	240 (13.5)	476 (13.6)
K50.1—large intestine	163 (9.5)	168 (9.4)	331 (9.5)
K50.8—both large and small intestine	226 (13.2)	556 (31.2)	782 (22.4)
K50.9—unspecified	1088 (63.5)	819 (45.9)	1,907 (54.5)
**Type of insurance, n (%)**			
Employee	1,235 (72.1)	1,293 (72.5)	2,528 (72.3)
Dependent—Adult	379 (22.1)	375 (21.0)	754 (21.6)
Dependent—Children	99 (5.8)	115 (6.4)	214 (6.1)
**CCI Components, n (%)**			
Peptic ulcer disease	745 (43.5)	928 (52.0)	1,673 (47.9)
Chronic pulmonary disease	670 (39.1)	670 (37.6)	1,340 (38.3)
Mild liver disease	413 (24.1)	448 (25.1)	861 (24.6)
Any malignancy, including lymphoma and leukemia, except malignant neoplasm of skin	177 (10.3)	137 (7.7)	314 (9.0)
Rheumatic disease	111 (6.5)	131 (7.3)	242 (6.9)
Congestive heart failure	104 (6.1)	68 (3.8)	172 (4.9)
Cerebrovascular disease	86 (5.0)	51 (2.9)	137 (3.9)
Diabetes without chronic complication	48 (2.8)	42 (2.4)	90 (2.6)
Renal disease	41 (2.4)	49 (2.7)	90 (2.6)
Peripheral vascular disease	110 (6.4)	47 (2.6)	157 (4.5)
**CCI score**			
Mean (SD)	1.8 (2.0)	1.6 (1.7)	1.7 (1.9)
Median (IQR)	1.0 (0.0–2.0)	1.0 (1.0–2.0)	1.0 (0.0–2.0)

**Abbreviations**: CCI: Charlson-comorbidity Index; CD: Crohn’s Disease; IQR: Interquartile range; SD: Standard Deviation

*Clinical characteristics of patients were measured throughout patient’s enrolment in JMDC.

### Healthcare resource utilization

Biologic initiation was associated with a reduction in the mean (SD) number of inpatient visits per patient/month from 0.4 (1.0) to 0.2 (0.2), mean (SD) length of stay (LOS) from 5.3 (15.1) days to 2.5 (3.9) days, and mean (SD) number of outpatient visits per patient/month from 1.9 (4.3) to 1.1 (1.0). Similarly, biologic initiation was associated with a reduction in the overall number of hospitalizations and surgical procedures, but an increase in requirement of enteral nutrition (Table 2).

**Table 2:** CD-related HRU one year before and one year after biologic index date.

**Number of visits per CD patient/ month**	**Before biologics (n = 1,783)**	**After biologics (n = 1,783)**
**Inpatient visits**		
Mean (SD)	0.36 (0.97)	0.17 (0.17)
Median (IQR)	0.16 (0.08–0.25)	0.08 (0.08–0.16)
**Length of stay, days**		
Mean (SD)	5.34 (15.15)	2.46 (3.93)
Median (IQR)	1.89 (0.90–3.87)	1.15 (0.47–2.71)
**Outpatient visits**		
Mean (SD)	1.88 (4.27)	1.14 (0.98)
Median (IQR)	0.99 (0.68–1.46)	0.99 (0.74–1.32)
**Number of events**	**Before biologics (n = 1,783)**	**After biologics (n = 1,783)**
Hospitalization	578	558
Surgical procedures	308	184
Enteral nutrition	446	499

**Abbreviations**: CD: Crohn’s Disease; HRU: Healthcare Resource Utilization; IQR: Interquartile range; SD: Standard Deviation.

Table 3 shows the HRU 12, 24, and 36 months after biologic initiation. The 24 and 36 months refers to the time passed from the previous evaluation timepoint i.e. month 13 to 24 and month 25 to 36, respectively. Mean (SD) number of inpatient visits per patient/month were similar across all three time periods (0.14 [0.11] vs. 0.15 [0.13] vs. 0.15 [0.13]), while mean (SD) LOS decreased from 2.1 (2.8) days after 12 months to 1.8 (2.7) days after 24 months and increased to 2.2 (3.6) days after 36 months. Mean (SD) number of outpatient visits per patient/month reduced slightly from 1.1 (0.51) after 12 months to 1.0 (0.55) and 1.0 (0.53) after 24 and 36 months, respectively.

**Table 3:** CD-related HRU 12 months, 24 months 36 months after biologic index date.

Number of visits per CD patient/ month	12 months after biologic index date (n = 1,363)	24 months after biologic index date (n = 1,100)	36 months after biologic index date (n = 851)
**Inpatient visits**			
Mean (SD)	0.14 (0.11)	0.15 (0.13)	0.15 (0.13)
Median (IQR)	0.08 (0.08–0.16)	0.08 (0.08–0.16)	0.08 (0.08–0.16)
**Length of stay, days**			
Mean (SD)	2.15 (2.85)	1.78 (2.73)	2.20 (3.65)
Median (IQR)	1.23 (0.49–2.51)	0.82 (0.33–1.97)	0.82 (0.41–2.71)
**Outpatient visits**			
Mean (SD)	1.08 (0.51)	1.04 (0.55)	1.02 (0.53)
Median (IQR)	0.99 (0.74–7.23)	0.90 (0.66–1.23)	0.90 (0.66–1.15))

The 24 and 36 months refers to the time passed from the previous evaluation timepoint i.e. month 13 to 24 and month 25 to 36, respectively.

**Abbreviations**: CD: Crohn’s Disease; HRU: Healthcare Resource Utilization; IQR: Interquartile range; SD: Standard Deviation.

### Costs

Biologic initiation was associated with a reduction in mean (SD) per patient/month inpatient costs from ¥276,164 (782,751) to ¥152,073 (357,579) and outpatient costs from ¥207,982 (840,357) to ¥204,157 (374,506). However, biologic initiation was associated with an increase in mean (SD) per patient/month pharmacy costs from ¥145,685 (739,873) to ¥245,894 (433,730) which led to an increase in total costs from ¥467,483 (1,700,960) to ¥497,289 (789,378) (Table 4).

**Table 4:** Direct costs 12 months before and after biologic initiation.

Cost per CD patient/ month (¥)	12 months before biologics (n = 1,783)	12 months after biologics (n = 1,783)
**Inpatient**		
Mean (SD)	276,164 (782,751)	152,073 (357,579)
Median (IQR)	99,122 (48,406–211,180)	75,674 (33,372–168,414)
**Outpatient**		
Mean (SD)	207,982 (840,357)	204,157 (374,506)
Median (IQR)	27,547 (10,807–97,879)	183,219 (89,529–247,517)
**Pharmacy**		
Mean (SD)	145,685 (739,873)	245,894 (433,730)
Median (IQR)	2,568 (1,085–13,231)	183,400 (155,623–241,753)
**Total costs**		
Mean (SD)	467,483 (1,700,960)	497,289 (789,378)
Median (IQR)	98,724 (25,316–312,995)	397,200 (299,527–539,531)

**Abbreviations**: CD: Crohn’s Disease; IQR: Interquartile range; SD: Standard Deviation.

Biologic initiation was associated with a reduction in mean (SD) per patient/month inpatient, outpatient, pharmacy, and total costs from 12 months to 24 months. However, all the cost categories showed an increase from 24 to 36 months post-initiation (Table 5).

**Table 5:** Direct costs 12, 24, and 36 months after biologic initiation.

Cost per CD patient/ month (¥)	12 months after biologic index date (n = 1,363)	24 months after biologic index date (n = 1,100)	36 months after biologic index date (n = 851)
**Inpatient**			
Mean (SD)	123,189 (136,791)	106,559 (133,295)	126,418 (162,151)
Median (IQR)	79,219 (36,696–154,382)	57,542 (22,551–146,764)	57,106 (23,227–189,959)
**Outpatient**			
Mean (SD)	178,480 (109,500)	165,394 (108,567)	170,611 (107,930)
Median (IQR)	183,228 (94,818–239,407)	167,541 (76,648–226,998)	174,158 (102,970–235,308)
**Pharmacy**			
Mean (SD)	193,320 (71,326)	170,162 (73,702)	173,778 (79,007)
Median (IQR)	176,808 (153,713–222,501)	153,355 (132,907–201,113)	158,031 (132,113–214,273)
**Total costs**			
Mean (SD)	423,005 (182,367)	360,132 (181,575)	370,774 (190,366)
Median (IQR)	392,524.1 (305,029.3–510,890)	334,253.0 (250,486.9–464,974)	347,881 (257,216–472,191)

Patients who had records available for the complete 12, 24, or 36 months were included in the respective groups.

**Abbreviations**: CD: Crohn’s Disease; IQR: Interquartile range; SD: Standard Deviation.

## Discussion

This is the first study to describe the demographics, HRU, and direct costs before and after biologic initiation in CD-affected Japanese patients, using claims data. The major strengths of this study are database capture of the entire CD treatment pathway including all hospitalizations, outpatient visits, and pharmacy dispensing, resulting in inclusive HRU and cost estimation; and evaluation of HRU and direct medical costs in patients on ustekinumab, which was approved for treatment of CD in Japan in 2017. The higher prevalence of CD in males and individuals aged 18–50 years old in the current study is consistent with previous research conducted through national surveys and hospital or claims data analyses [4, 17, 18, 26]. Murakami et al [4] has posited smoking to be the reason for male predominance in CD, as smoking (both active and passive) are risk factors for CD [27, 28]. The current study also report comorbid conditions such as peptic ulcer, chronic pulmonary and mild liver disease that have direct impact on the claims cost and resource utilization during CD treatment. The baseline data of comorbid conditions also helps in data interpretation.

Biologic initiation was associated with a 11% increase in enteral nutrition. However, this could be attributed to a unique practice recommended in Japanese clinical treatment guidelines [8] wherein enteral nutrition is recommended for mild CD patients before starting conventional drug treatment, for moderate to severe patients to control food intake, for those who may not fully respond to biologic treatment, and for those who received surgery. Moreover, Japanese studies have reported satisfactory remission induction rate and maintenance rates with enteral nutrition [29]. Further studies are needed to understand the initiation of biologics and the increased burden of enteral nutrition in Japan.

Biologic initiation was associated with a reduction in inpatient visits by 53%, LOS by 54%, surgical procedures by 40%, inpatient costs by 45%, outpatient visits by 39%, and outpatient costs by 2%. However, biologic initiation was associated with an increase in pharmacy costs by 40% and total costs by 6%. Similarly, other studies in various healthcare systems have associated biologic initiation with a reduction in inpatient and outpatient visits and their associated costs; however, an increase in pharmacy costs, most likely due to biologic prescriptions, resulted in greater total direct costs compared with pre-biologic expenses [30–32]. Although the present study did not distinguish between biologic and other drug-related costs, Saito et al [18] analyzed patient data (2013–2016) from the JMDC database and reported the total CD-related costs to be ¥7,139,604,830, with anti-TNFα agents accounting for 59.5% of the total costs. Studies from the Netherlands [33] and United States [34, 35] have also reported biologic-related costs to be a major driver of total costs.

We also evaluated HRU and direct costs 12, 24, and 36 months after biologic initiation. Here, compared to 12-month data, HRU and costs seemed to be similar or lower after 24 months of follow-up. However, HRU and direct costs after 36 months of follow-up were higher than the 24-month data, suggesting that some patients undergoing biologic treatment may not respond optimally after 24 months of treatment, and hence may have to switch treatment or undergo surgery to manage CD. This seems plausible since previous research has reported a reduction in persistence with biologics as time passes, with a wide range in the rate of non-persistence due to treatment discontinuation or switching [36–39].

Japanese clinical guidelines recommend biologics for treatment of moderate to severe and severe CD [7, 40]. Hence, real-world treatment practice may reflect this recommendation, as a higher proportion of patients on biologics in the current study had CD in both the small and large intestine compared with patients not on biologics (31.2% vs. 13.2%). Complications such as fistulae have an adverse impact on a patient’s health-related quality of life (HRQoL) [7] and also result in greater median costs than those without fistulae ($10,868 vs. $6,268), primarily due to hospitalization and surgery [41]. Hence, optimally managing fistulae quickly is required. Infliximab has been reported to significantly reduce hospitalizations, LOS, and surgeries (P<0.05) in patients with fistulizing CD compared with placebo treatment [42]. Moreover, a Markov model by Lindsay et al [43] showed an eight-week scheduled maintenance treatment with infliximab to be cost-effective in patients with active luminal or fistulizing CD. Thus, usage of biologics early could potentially reduce disease progression and HRU as well as translate into long-term cost savings.

Patients with inflammatory bowel disease (IBD) experience lower HRQoL; higher absenteeism, presenteeism, and work and activity impairment; higher HRU; and higher direct and indirect costs compared with the general population [17, 44–47]. However, comparing IBD with other diseases may reveal the true burden of IBD on society. Although cancer is the leading cause of death in Japan (death rate 298.3 per 100,000 people) [48], cancer-affected adults have reported higher HRQoL and lower work and activity impairment than IBD-afflicted adults in Japan [49]. Moreover, the total mean per-patient/month costs for CD are also higher than another intractable disease such as systemic lupus erythematosus (¥497,289 [current study] vs. ¥84,751 [Miyazaki et al] [20]), thus highlighting the huge burden of IBD on a patient’s daily life. Biologics have been reported to induce and maintain clinical remission in Japanese patients with moderate to severe CD [50] as well as reduce the burden of IBD by improving HRQoL, reducing work and activity impairment, and decreasing HRU across varying healthcare systems [30, 50–56]. Although biologic initiation is associated with higher direct costs, as observed in our study, biologic initiation may reduce indirect costs due to its positive impact on work productivity. This is especially relevant in Japan where patients with IBD have reported facing difficulties at work due to IBD, resulting in poor performance, lack of career advancement, and workplace alienation [57]. Moreover, patients with IBD have a higher unemployment rate compared with the general population (12.3% vs. 3.2%) [58]. Therefore, biologics may help to increase work productivity as well as reduce the unemployment rate, resulting in lower indirect costs. Unfortunately, the effect of biologics on indirect costs in Japan has not been evaluated. Further research could shed light on this aspect.

### Limitations

This study has several limitations. First, the JMDC database does not include clinical examination results or physician-assessed severity, which could have enabled stratification of treatment based on disease severity. Second, as the database records insurance claims for employees of medium- to large-scale companies and their dependents only, this study could not assess data of patients who are not covered in the database. Third, as the database is linked to employment status, the elderly are underrepresented in the database. Therefore, the results of this study may not be applicable to every age group. Moreover, since cost data is variable in nature, a larger sample size may be required. A limitation of assessing costs in pre- and post- study designs is that costs incurred due to failure of treatment A but before initiating treatment B, are poorly assessed. Although patients with severe disease often incur higher costs [20], we reported mean cost per patient/month since we were unable to assess treatment costs based on disease severity. However, it is possible to provide an expenditure estimate for payers since the JMDC allows for projection of nationwide totals [59]. A major limitation of the study is the lack of evaluation of work productivity and activity impairment and associated indirect costs, which have been reported to be high in Japanese patients with CD [17]. We were also unable to capture the costs for patients who require long-term care as these costs are not captured in the database. Evaluation of the impact of biologics on indirect costs may reveal the true effect of biologics on society. Finally, due to the cross-sectional nature of the data, causal inferences cannot be made from the results.

## Conclusion

Our study demonstrated that biologic therapy in patients with CD was associated with a reduction in HRU and related costs, and a slight increase in total pharmacy costs compared with their treatment period before biologic therapy. Use of effective biologic therapies for CD treatment can gradually reduce the economic burden on government that provides financial assistance to patients with CD in Japan. Furthermore, evaluation of indirect costs in the future may reveal the true impact of biologics on payers, and may help in appropriate allocation of resources to reduce the burden on both patients and payers.

## Supporting information

S1 Table Pharmacological agents. (DOCX)

S2 Table Enteral nutrition procedures. (DOCX)

S3 Table Surgical procedures. (DOCX)
