# Supplementary material for: Evaluation of economic burden with biologic treatments in Crohn’s disease patients: A mirror image study using an insurance database in Japan
Source: PLoS One. 2021 Jul 19;16(7):e0254807. doi: 10.1371/journal.pone.0254807 (PMC8289035; doi:10.1371/journal.pone.0254807)
Supplement: S1 Table — (DOCX) [file pone.0254807.s001.docx]

**S1** **Table: Pharmacological agents**

| **ATC codes** | **Name** | **Frequency of administration** |
| --- | --- | --- |
| **Biologic Agents** | | |
| L04AB02 | Infliximab | Induction dose: Intravenously 5mg/kg at week 0, 2, and 6, and subsequently 8 weeks thereafter  [or can be shortened to every 4 weeks with diminished effects] |
| L04AB04 | Adalimumab | Induction dose: Subcutaneously 160mg as at week 0  Second dose: Subcutaneously 80mg at week 2  Maintenance: Subcutaneously 40mg every 2 weeks from week 4 onwards  [or can be increased to 80mg with diminished effect] |
| L04AC05 | Ustekinumab | Induction dose: Intravenously  ≤55 kg: 260 mg  >55 kg to 85 kg: 390 mg  >85 kg: 520 mg  Maintenance: Subcutaneously 40mg every 8 to 12 weeks  [can be increased to every 8 weeks with diminished effect] |
| **5-Aminosalicylic Acids** | | |
| A07EC01 | sulfasalazine | Daily |
| A07EC02 | mesalazine | Daily |
| **Corticosteroids** | | |
| H02AB04 | methylprednisolone | Daily/ short courses |
| H02AB09 | hydrocortisone | Daily |
| A07EA01/ H02AB06 | prednisolone | Daily |
| A07EA03/ H02AB07 | prednisone | Daily |
| A07EA06 | budesonide | Daily |
| **Immunomodulating Drugs** | | |
| L01BA01/ L04AX03 | methotrexate | Weekly |
| L01BB02 | mercaptopurine | Daily |
| L04AX01 | azathioprine | Daily |
| L04AD01 | ciclosporin | Daily |
| L04AD02 | tacrolimus | Daily |
| L04AA06 | mycophenolate | Daily |
