# Supplementary material for: Evaluation of economic burden with biologic treatments in Crohn’s disease patients: A mirror image study using an insurance database in Japan
Source: PLoS One. 2021 Jul 19;16(7):e0254807. doi: 10.1371/journal.pone.0254807 (PMC8289035; doi:10.1371/journal.pone.0254807)
Supplement: S2 Table — (DOCX) [file pone.0254807.s002.docx]

**S2 Table:** **Enteral nutrition procedures**

| **Standardized_ procedure_id** | **Procedure_code** | **Description of Procedure** |
| --- | --- | --- |
| 190170770 | A108 | Additional fee for nutrition management |
| 190120410 | A233 | nutrition management fee |
| 190136810 | A233-2 | nutrition support team fee |
| 113001010 | B001 | diet and nutrition guidance fee for outpatient |
| 113003310 | B001 | diet and nutrition guidance fee for inpatient |
| 113003410 | B001 | diet and nutrition guidance fee in group |
| 113014810 | B001 | diet and nutrition guidance fee for inpatient [1] |
| 113017410 | B001 | diet and nutrition guidance fee for outpatient (first time) |
| 113017510 | B001 | diet and nutrition guidance fee for outpatient (second time or after) |
| 113017610 | B001 | diet and nutrition guidance fee for inpatient [1], first time |
| 113017710 | B001 | diet and nutrition guidance fee for inpatient [1], second time |
| 111000470 | B001-2-3 | nutrition guidance fee for infant |
| 114004210 | C104 | guidance fee for in-home parenteral nutrition |
| 114005110 | C104 | additional fee for infusion set <guidance fee for in-home parenteral nutrition> |
| 114006710 | C104 | additional fee for infusion pump <guidance fee for in-home parenteral nutrition> |
| 114005110 | C160 | infusion set fee for home parenteral nutrition |
| 114005210 | C162 | feeding tube set fee for home eternal nutrition |
| 130009070 | K618 | implantable parenteral nutrition catheter placement (extremity) |
| 130009170 | K618 | implantable parenteral nutrition catheter placement (head, neck and other sites) |
