# Supplementary material for: Evaluation of economic burden with biologic treatments in Crohn’s disease patients: A mirror image study using an insurance database in Japan
Source: PLoS One. 2021 Jul 19;16(7):e0254807. doi: 10.1371/journal.pone.0254807 (PMC8289035; doi:10.1371/journal.pone.0254807)
Supplement: S3 Table — (DOCX) [file pone.0254807.s003.docx]

**S3 Table: Surgical procedures**

| **Standardized_ procedure_id** | **Description of Procedure** |
| --- | --- |
| 150157810 | abdominal wall abscess incision |
| 150157910 | abdominal wall fistula surgery (confined to abdominal wall) |
| 150160010 | exploratory laparotomy |
| 150160310 | localized abdominal abscess surgery (periappendicular abscess) |
| 150160410 | localized abdominal abscess surgery (other) |
| 150160610 | pelvic extraperitoneal abscess incision and drainage |
| 150160810 | acute generalized peritonitis surgery |
| 150162310 | malignant retroperitoneal tumor surgery |
| 150164410 | endoscopic gastric/duodenal polypectomy / mucosal resection, other polypectomy / mucosal resection |
| 150164850 | endoscopic gastrointestinal hemostasis |
| 150165050 | endoscopic removal of esophageal and gastric foreign body |
| 150168010 | gastrectomy (malignant tumor surgery) |
| 150169950 | malignant gallbladder tumor surgery (confined to gallbladder, including lymph node dissection) |
| 150171310 | gastroenterostomy (including Braun's anastomosis) |
| 150171610 | gastrostomy (including percutaneous endoscopic gastrostomy) |
| 150171810 | pyloroplasty (including extramucosal pyloromyotomy) |
| 150174910 | endoscopic removal of biliary stone (with biliary lithotripsy) |
| 150175310 | endoscopic biliary dilatation |
| 150175410 | endoscopic papillotomy (papillary sphincterotomy only) |
| 150178110 | pancreatic body and tail tumor resection (distal pancreatectomy, including tumor resection) (simultaneous resection of spleen) |
| 150180210 | intestinal adhesion surgery |
| 150180350 | intestinal obstruction surgery (intestinal adhesion surgery) |
| 150180650 | intestinal obstruction surgery (small intestine resection) (resection other than malignant tumor) |
| 150181210 | small intestine resection (resection other than malignant tumor) |
| 150181710 | colectomy (small range resection) |
| 150181810 | colectomy (hemicolectomy) |
| 150181910 | colectomy (total colectomy / subtotal colectomy / malignant tumor surgery) |
| 150183110 | colon tumor resection (including ileocecal tumor resection) |
| 150183410 | endoscopic colon polypectomy/mucosal resection (other) |
| 150183650 | fiberscopic colorectal polypectomy |
| 150184110 | intestinal anastomosis |
| 150184510 | Colostomy |
| 150184710 | enterostenosis incision suture |
| 150185210 | closure of small intestinal fistula (without intestinal resection) |
| 150185310 | closure of small intestinal fistula (with intestinal resection) |
| 150185410 | closure of colonic fistula (without intestinal resection) |
| 150185510 | closure of colonic fistula (with intestinal resection) |
| 150186110 | colostomy (with laparotomy) |
| 150186210 | colostomy (other) |
| 150186510 | perirectal abscess incision |
| 150186810 | rectal tumor resection (transanal) |
| 150187110 | rectal resection/amputation (resection) |
| 150187210 | rectal resection/amputation (amputation) |
| 150187510 | repair of rectal stenosis |
| 150189810 | perianal abscess incision |
| 150189910 | anal fistula radical surgery (simple) |
| 150190010 | anal fistula radical surgery (complicated) |
| 150190150 | high rectal fistula surgery |
| 150191810 | anoplasty (repair of anal stenosis) |
| 150191910 | anoplasty (rectal mucosal prolapse repair) |
| 150245410 | rectal resection/amputation (lower anterior resection) |
| 150254410 | endoscopic biliary stent placement |
| 150263950 | endoscopic small intestinal and colonic hemostasis |
| 150271550 | laparoscopic enterolysis |
| 150271950 | laparoscopic small intestine resection |
| 150276410 | endoscopic gastric/duodenal polypectomy / mucosal resection, early malignant tumor mucosal resection |
| 150276810 | laparoscopic cholangiotomy for gallstone removal (including cholecystectomy) |
| 150277810 | laparoscopic colectomy |
| 150279970 | additional fee for laparoscopic surgery <closed circuit general anesthesia> |
| 150285010 | endoscopic colon polypectomy/mucosal resection (long diameter, shorter than 2 cm) |
| 150293310 | small intestinal/colonic stenosis dilation (endoscopy) |
| 150296710 | endoscopic papillotomy (with biliary lithotripsy) |
| 150297310 | small intestine resection (malignant tumor surgery) |
| 150323210 | local gastric resection |
| 150323510 | laparoscopic gastrectomy (malignant tumor surgery) |
| 150324910 | laparoscopic malignant colon tumor resection |
| 150325010 | endoscopic removal of colonic foreign body |
| 150325210 | laparoscopic rectal resection |
| 150337710 | laparoscopic colectomy (total colectomy / subtotal colectomy) |
| 150337810 | laparoscopic rectal resection/amputation (lower anterior resection) |
| 150337910 | laparoscopic rectal resection/amputation (amputation) |
| 150339010 | surgery support fee (navigation) |
| 150341450 | endoscopic papillary dilatation |
| 150347510 | percutaneous abdominal abscess drainage |
| 150348510 | endoscopic colorectal polypectomy (long diameter, 2 cm or longer) |
| 150361410 | laparoscopic exploratory laparotomy |
| 150361610 | laparoscopic diffuse peritonitis surgery |
| 150362310 | endoscopic nasobiliary drainage (ENBD) |
| 150362510 | endoscopic removal of biliary stone (other) |
| 150363610 | endoscopic pancreatic stent placement |
| 150363710 | laparoscopic small intestine resection (malignant tumor) |
| 150363810 | total colonic/rectal resection and ileoanal anastomosis |
| 150371290 | additional fee for holiday [1] [surgery] |
| 150371390 | additional fee for after hour [1] [surgery] |
| 150371490 | additional fee for late night [1] [surgery] |
| 150371590 | additional fee for after hour surgery at special medical institution [1] [surgery] |
| 150377610 | laparoscopic local gastric resection (combined with endoscopic treatment) |
| 150378110 | removal of gastrostomy tube |
| 150388670 | additional fee for balloon enteroscopy <endoscopic removal of biliary stone> |
| 150389610 | laparoscopic colostomy |
| 190181210 | short stay for surgery basic fee [3-s] K721, endoscopic colon polypectomy/mucosal resection [1], long diameter, shorter than 2 cm |
| 190181410 | short stay for surgery basic fee [3-t] K721, endoscopic colon polypectomy/mucosal resection [2], long diameter, 2 cm or longer |
| 999900418 | [J] anal fissure/ulcer radical surgery |
